# Contrast-enhanced small-animal PET/CT in cancer research: strong improvement of diagnostic accuracy without significant alteration of quantitative accuracy and NEMA NU 4–2008 image quality parameters

**DOI:** 10.1186/2191-219X-3-5

**Published:** 2013-01-17

**Authors:** Charline Lasnon, Elske Quak, Mélanie Briand, Zheng Gu, Marie-Hélène Louis, Nicolas Aide

**Affiliations:** 1 Normandie Université, Caen, 14000, France; 2 BioTICLA Team, EA 4656, IFR 146, François Baclesse Cancer Centre, 3 Avenue Général Harris, Caen Cedex 5, 14076, France; 3 Nuclear Medicine Department, François Baclesse Cancer Centre, 3 Avenue Général Harris, Caen Cedex 5, 14076, France; 4 Biomedical Physics Program, UCLA, Los Angeles, CA, 90024, USA

**Keywords:** Small-animal PET/CT, Contrast media, NEMA NU 4–2008, Tumor-bearing rodents, Quantification

## Abstract

**Background:**

The use of iodinated contrast media in small-animal positron emission tomography (PET)/computed tomography (CT) could improve anatomic referencing and tumor delineation but may introduce inaccuracies in the attenuation correction of the PET images. This study evaluated the diagnostic performance and accuracy of quantitative values in contrast-enhanced small-animal PET/CT (_CE_PET/CT) as compared to unenhanced small animal PET/CT (_UE_PET/CT).

**Methods:**

Firstly, a NEMA NU 4–2008 phantom (filled with ^18^F-FDG or ^18^F-FDG plus contrast media) and a homemade phantom, mimicking an abdominal tumor surrounded by water or contrast media, were used to evaluate the impact of iodinated contrast media on the image quality parameters and accuracy of quantitative values for a pertinent-sized target. Secondly, two studies in 22 abdominal tumor-bearing mice and rats were performed. The first animal experiment studied the impact of a dual-contrast media protocol, comprising the intravenous injection of a long-lasting contrast agent mixed with ^18^F-FDG and the intraperitoneal injection of contrast media, on tumor delineation and the accuracy of quantitative values. The second animal experiment compared the diagnostic performance and quantitative values of _CE_PET/CT versus _UE_PET/CT by sacrificing the animals after the tracer uptake period and imaging them before and after intraperitoneal injection of contrast media.

**Results:**

There was minimal impact on IQ parameters (%SD_unif_ and spillover ratios in air and water) when the NEMA NU 4–2008 phantom was filled with ^18^F-FDG plus contrast media. In the homemade phantom, measured activity was similar to true activity (−0.02%) and overestimated by 10.30% when vials were surrounded by water or by an iodine solution, respectively. The first animal experiment showed excellent tumor delineation and a good correlation between small-animal (SA)-PET and *ex vivo* quantification (*r*^2^ = 0.87, *P* < 0.0001). The second animal experiment showed a good correlation between _CE_PET/CT and _UE_PET/CT quantitative values (*r*^2^ = 0.99, *P* < 0.0001). Receiver operating characteristic analysis demonstrated better diagnostic accuracy of _CE_PET/CT versus _UE_PET/CT (senior researcher, area under the curve (AUC) 0.96 versus 0.77, *P* = 0.004; junior researcher, AUC 0.78 versus 0.58, *P* = 0.004).

**Conclusions:**

The use of iodinated contrast media for small-animal PET imaging significantly improves tumor delineation and diagnostic performance, without significant alteration of SA-PET quantitative accuracy and NEMA NU 4–2008 IQ parameters.

## Background

Small-animal positon emission tomography (SA-PET) imaging is a powerful tool in performing preclinical studies in tumor-bearing rodents and is being increasingly used to evaluate metabolic response to treatment, particularly within the framework of new drug development [1,2]. Animal models include abdominal tumors that arise in transgenic animals or tumors that arise after engraftment of exogenous malignant cells.

In abdominal tumor models, a moderate-to-high physiological uptake in the gut is observed with many tracers, including ^18^F]fluorodeoxyglucose (^18^F-FDG) and ^18^F]fluorothymidine (^18^F-FLT), which may hamper tumor detection. Moreover, anatomical landmarks are lacking on PET images alone. In this setting, correlative computed tomography (CT) acquisition is useful to improve the localization of tracer uptake and provides faster and more accurate attenuation correction than attenuation correction obtained with external gamma sources [3]. However, an unenhanced CT scan does not allow delineation of tumors from the surrounding organs as both have similar CT attenuation characteristics [4,5]. This drawback is particularly an issue in nude mice and nude rats, which lack abdominal fat. The use of iodinated contrast agents solves this problem. Iodinated agents that are typically used in the clinical setting cannot be used for intravenous injections in rodents because microCT devices are not fast enough to image animals before the contrast media is cleared from the blood. Therefore, dedicated iodinated contrast agents have been developed for preclinical research. Two long-lasting intravascular contrast media agents, eXIA 160XL (Binitio Biomedical, Inc., Ottawa, Canada) and Fenestra VC (Advanced Research Technologies Inc., Saint-Laurent, Quebec, Canada) [6-9], are commercially available. eXIA 160XL is a single-phase contrast agent that provides peak contrast enhancement in the spleen, liver, and vessels within the first hour after injection, while Fenestra VC is a dual-phase contrast agent that provides blood-pool enhancement for more than 2 h after intravenous injection followed by liver enhancement. Also available is Fenestra LC (Advanced Research Technologies Inc.), a contrast agent offering liver and spleen enhancement for durations reported to be as long as 7 days in mice. In addition, the clinically used iodinated agents can be injected by the intraperitoneal route to improve delineation of abdominal tumors [10].

To our knowledge, studies comparing the diagnostic performance of unenhanced SA-PET/CT versus contrast-enhanced SA-PET/CT with protocols including one or more of the contrast agents described above are lacking. Moreover, clinical PET/CT studies have shown that contrast agents may result in overestimation of standardized uptake values on images corrected for attenuation with attenuation maps derived from CT [11-15].

The aim of this study was to perform a comprehensive evaluation of the diagnostic performance and the accuracy of SA-PET quantitative values in contrast-enhanced versus unenhanced SA-PET/CT by phantom and animal studies in abdominal tumor-bearing rats and mice. For that purpose, we used the NEMA NU 4–2008 image quality phantom, which provides a standardized assessment of the overall image quality and accuracy of attenuation and scatter corrections [16]; we also used homemade phantoms mimicking tumors surrounded by contrast media. In addition, we scanned a large cohort of animals with various contrast media protocols to evaluate both the diagnostic and quantitative accuracies of contrast-enhanced SA-PET/CT (_CE_PET/CT) versus unenhanced SA-PET/CT (_UE_PET/CT).

## Methods

### SA-PET/CT data acquisition and reconstruction parameters

SA-PET/CT examinations were performed on an Inveon SA-PET/CT (Siemens Medical Solutions, Knoxville, TN, USA). First, SA-CT images were acquired in approximately 10 min using 80 keV and 500 μA. Then PET acquisitions were performed using energy and coincidence timing windows of 350 to 650 keV and 3.4 ns, respectively. Emission scan duration was 20 min for phantom acquisitions, as recommended by the NEMA NU 4–2008 standards, and 15 min for animal scans. When the animal was imaged more than once, the duration of the subsequent acquisition was increased to account for ^18^F decay.

Reconstructions were performed using a three-dimensional maximum *a posteriori* (MAP) reconstruction with a 128 × 128 transaxial image matrix size. Three-dimensional ordered subset expectation maximization (OSEM-3D)/MAP was used with 2 OSEM-3D iterations and 18 MAP iterations with the β parameter set to 0.2. For NEMA NU 4 phantom studies, reconstructions were performed (a) with attenuation and scatter corrections, (b) with attenuation correction but without scatter correction, and (c) with neither attenuation nor scatter correction. For animal studies, data were corrected for attenuation and scatter events.

#### Phantom studies

Phantom studies were carried out with the NEMA NU 4–2008 image quality phantom. This phantom has the following features: a main fillable cylindrical chamber of 30-mm diameter and 30-mm length; a solid part with five fillable rods drilled through (at 7 mm from the center) with diameters of 1, 2, 3, 4, and 5 mm, respectively, and 20 mm in length; and a part with two cold cylindrical chambers 15 mm in length and 8 mm in diameter (one filled with non-radioactive water and the other with air). A more detailed description can be found elsewhere [17]. The image quality phantom was filled either with an ^18^F-FDG solution (diluted with pure water) or with an ^18^F-FDG solution containing iohexol at a concentration of 100 mg iodine (I)/mL, representing the highest concentration from our preclinical protocols.

Radioactivity at the beginning of the emission scan was 3.7 MBq ± 5%. The NEMA NU 4–2008 phantom was scanned twice for each situation.

Moreover, a homemade phantom was used to evaluate the impact of high Hounsfield densities on the accuracy of quantitative values for a pertinent-sized target. This phantom was designed to mimic tumors surrounded by water or intraperitoneal contrast media: small tubes (volume 2 mL, diameter 10 mm) were filled with an ^18^F-FDG-containing solution and placed at the center of a 20-mL syringe (diameter 18 mm) filled either with water or with a solution of iohexol (100 mg I/mL) inserted into a 60-mL syringe (diameter 27 mm). Syringes were consecutively scanned four times, with ^18^F-FDG concentrations ranging from 0.38 to 0.87 MBq/mL.

For each acquisition with contrast media, the phantom and vials were gently shaken immediately before the start of the CT scan in order to keep the iodine solution homogeneous. Preliminary studies (data not shown) have demonstrated that no sedimentation of iodine contrast media occurred within 45 min following the preparation of a phantom containing contrast material. Thus, it was not necessary to shake the phantom a second time after the CT had been performed, since overall, the acquisition time for a phantom SA-PET/CT acquisition was not more than 35 min.

#### Animal experiments

The regional ethics committee approved the experiments. A total of 16 mice and 6 rats were used. Four-week-old nude mice and nude rats were intraperitoneally injected with human ovarian cancer cell lines (SKOV-3 and OVR cell lines, purchased from American Type Culture Collection). Animals were kept under pathogen-free conditions and fed and watered *ad libitum* except on the day of SA-PET examination when a 6-h fasting period was maintained prior to the tracer injection.

Animals were injected with ^18^F-FDG in the tail vein under general anesthesia. Mean administered activity was 10 MBq for mice and 40 MBq for rats. Before, during, and after the ^18^F-FDG injections, animals were kept under an infrared light to minimize brown fat visualization. For general anesthesia, heated inhaled isoflurane was administered with an anesthesia device dedicated to small animals (Minerve, France). Mice were imaged in groups of four using a customized bed scanner. Technologic issues related to multiple mice imaging have been previously reported [18,19] and have been recently discussed in detail and are beyond the scope of this article [20].

**Figure 1:**
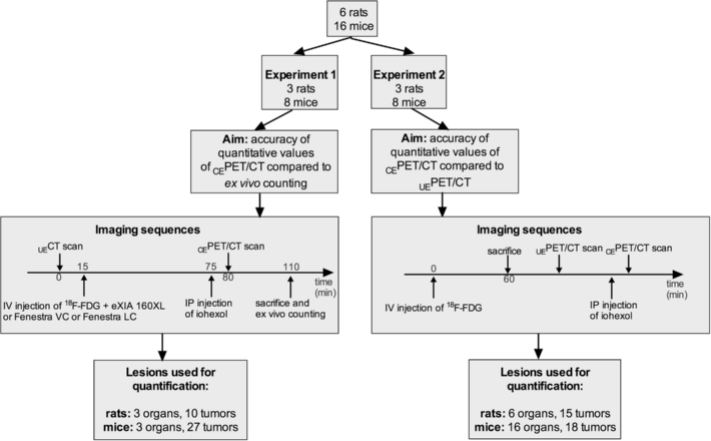
**Flow chart of animal experiments.** Number of animals used in each experiment, timeline for tracer and iodinated contrast media injections, and number of tumors and organs used for quantification are displayed. Volumes injected for IV contrast media (eXIA 160XL, Fenestra VC, or Fenestra LC) ranged from 0.10 to 0.15 mL in mice and were 0.80 mL in rats, and those for IP injections (iohexol at a concentration of 100 mg I/mL) were 1 mL in mice and 3 mL in rats.

Figure 1 summarizes the animal experiments that were performed. In the first experiment, designed to study the impact of various contrast media protocols on the accuracy of quantitative values as compared to *ex vivo* counting, 11 animals were sacrificed immediately after the PET acquisition. In the second experiment, aimed at comparing the diagnostic performance and quantitative values of _CE_PET/CT and _UE_PET/CT acquisitions, 11 animals were sacrificed after the tracer uptake period to stop tracer uptake that may have occurred in between the two series of SA-PET/CT examinations.

### *Ex vivo* counting

In the first experiment, immediately after the PET examination, tumors and organs were harvested. Tissue samples were weighed with a precision scale (±0.01 mg). Tumor radioactivity was counted for 2 min in a cylinder-well counter (Cobra II, Packard, GMI, Inc., MN, USA) and corrected for instrument efficiency and decay. Counts per minute were converted to becquerel and normalized for sample weight, assuming a density of 1 g/mL.

### Calibration and cross calibration

The SA-PET/CT system was calibrated according to the manufacturer's guidelines, by imaging a ^68^Ge cylinder phantom. A cross calibration among the SA-PET/CT system, the dose calibrator, and the gamma counter was performed. A 10 MBq ^18^F-FDG solution (as assessed by the dose calibrator) was used to fill a vial with an exact known volume, which resulted in a solution with an exactly known concentration. This solution was used to fill a cylinder phantom that was scanned for 20 min on the SA-PET/CT scanner. Three samples of this solution (0.5 mL) were also drawn up with a calibrated pipette to be counted in the gamma counter. A large volume of interest (VOI) was used to determine the mean activity concentration as assessed by the SA-PET/CT scanner. Cross-calibration factors were then derived and used to synchronize counts/measurements for the three pieces of equipment.

### Data analysis

#### Phantom studies

The following NEMA NU 4–2008 parameters were determined for each configuration and each reconstruction: (1) image noise was defined by the percentage standard deviation (%SD_unif_) in a VOI (22.5-mm diameter, 10-mm length) drawn over the center of the uniform region, and (2) spillover ratios in water (SOR_wat)_ and in air (SOR_air_) were determined by the ratio of the mean activity concentration in the VOIs defined in each cold region (water and air, 4-mm diameter, encompassing the central 7.5 mm in length in the axial direction) divided by the mean activity concentration of the uniform area. Data were processed with AMIDE [21]. For acquisitions involving the homemade phantoms filled with either an ^18^F-FDG solution or a mixture of ^18^F-FDG plus iohexol, cylindrical VOI (7.5-mm diameter, 10-mm length) was drawn, and the ratios between true activity and measured activity were recorded.

#### Animal studies

In the first experiment, to evaluate the accuracy of quantitative values extracted from _CE_PET/CT, 3D VOIs were drawn over the tumors, thanks to an isocontour with a threshold set so that the VOI matched the apparent tumor volume on the CT component of PET/CT images. Even when a discordance occurred between PET metabolic volume and CT volume, the VOI was drawn according to CT images so that PET/CT images could be compared to *ex vivo* counting for which the entire tumor was harvested, irrespective of the presence of non-viable areas.

In the second experiment, the impact of the use of contrast media on tumor detection and accuracy of quantitative data was assessed. The _CE_PET/CT and _UE_PET/CT data were randomly interpreted 4 weeks apart by a senior researcher with 4 years of experience in SA-PET/CT and 7 years of experience in SA-PET and a junior researcher. Observers, who were blinded to the animals' status (i.e., presence or absence of tumors within the abdomen), rated each abdominal focus using a 5-point scale (1, definitely benign; 2, probably benign; 3, indeterminate; 4, probably malignant; 5, definitely malignant). Each rating was compared to the results of the necropsy. Diagnostic performance of _CE_PET/CT and _UE_PET/CT was compared by means of receiver operating characteristic analysis [22]. Then, PET quantitative values for _UE_PET/CT and _CE_PET/CT were compared. For that purpose, the two sets of images were displayed side by side, and 3D VOI was determined by means of an isocontour, which was set so that (a) the VOI matched the apparent tumor volume on PET images and (b) the tumor volume was equal on both sets of PET data.

### Statistical analysis

The relationship between the radioactivity in animal tumors as determined by SA-PET/CT and by gamma counter was estimated using linear regression analysis. In addition to regression analysis, Bland-Altman plots were produced [23]. The same type of analysis was used to compare quantitative values extracted from _UE_PET/CT and _CE_PET/CT. Areas under the receiver operating characteristic curve for _CE_PET/CT versus _UE_PET/CT were compared according to the methodology of DeLong et al. [22].

## Results

### Impact of contrast media on image quality and accuracy of quantitative values

#### Phantom studies

**Figure 2:**
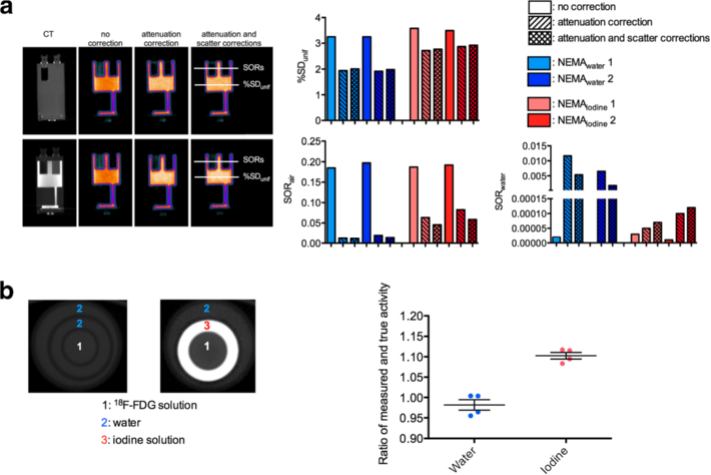
**Impact of high densities induced by iodine-based contrast media.** On NEMA NU 4 image quality phantom measurements (**a**) and SA-PET/CT quantitative values (**b**). (**a**) Representative coronal slices of the NEMA NU 4 image quality phantom at the level of the water- and air-filled cylinders are shown (left panel). Percentage standard deviation (%SD_unif_) and spillover ratios in water (SOR_wat_) and in air (SOR_air_) are shown in the right panel for the phantom filled with an ^18^F-FDG solution (blue) or a mixture of ^18^F-FDG and iodinated contrast media at a final concentration of 100 mg I/mL (red). PET data were reconstructed with both attenuation and scatter corrections, with only attenuation correction and with neither attenuation nor scatter correction. (**b**) Ratios of measured and true activity for ^18^F-FDG sources surrounded by water or by a combination of water and iodine layers (four consecutive measurements were performed; bars denote mean ± %SD). Transverse sections through the homemade phantom are shown, illustrating the different parts of the phantom: small tubes (diameter 10 mm) filled with an ^18^F-FDG-containing solution and placed at the center of a syringe (diameter 18 mm) filled either with water or with a solution of iohexol (100 mg I/mL) inserted into a second syringe (diameter 27 mm).

%SD_unif_ is a measure of signal to noise ratio and is in part affected by scatter and attenuation correction performance, while SOR_air_ and SOR_water_ are measures of scatter correction performance. As shown in Figure 2, changes for %SD_unif_ and SOR values when data were reconstructed with attenuation correction or with both scatter and attenuation corrections were slightly different for the water-filled and the iodine-filled NEMA phantoms. For uncorrected data, %SD_unif_, SOR_air_, and SOR_water_ were almost similar for all acquisitions (approximately equal to 3.4, 0.2, and 0, respectively). For data fully corrected for attenuation and scatter events, %SD_unif_ and SOR_air_ tended to be higher for the iodine-filled phantom (on average, 2.845 and 0.052, respectively) as compared to the water-filled phantom (on average, 1.995 and 0.013, respectively).

Additional phantom acquisitions were performed to evaluate the impact of high densities on the accuracy of quantitative values for a pertinent-sized target. For that purpose, a homemade phantom mimicking an abdominal tumor surrounded either by contrast media or by water was used (Figure 2). When vials were surrounded by water, measured activity was almost equal to true activity (−0.02%). When vials were surrounded by an iodine solution at a concentration similar to that intraperitoneally injected into rodents (100 mg I/mL), a mean overestimation of 10.30% was identified.

#### Animal studies

**Figure 3:**
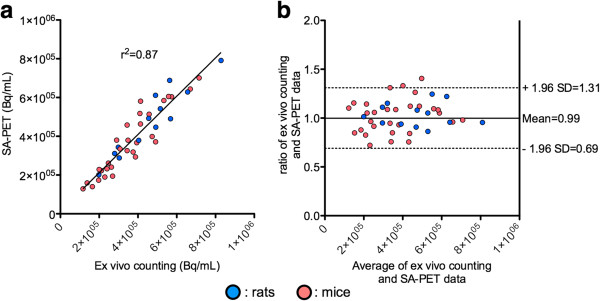
**Accuracy of contrast-enhanced SA-PET/CT quantitative values.** Linear regression (**a**) and Bland-Altman analysis (**b**) of SA-PET/CT quantitative values (mean VOI value) as compared to *ex vivo* counting. Nude mice and nude rats bearing abdominal tumors received contrast enhancement via intraperitoneal injection of iohexol (100 mg I/mL) and intravenous injection of long-lasting intravenous contrast media (eXIA 160XL or Fenestra VC) or hepatocyte-selective contrast media (Fenestra LC).

Figure 1 summarizes the imaging protocols used in mice and rats bearing abdominal tumors. The first experiment was designed to evaluate the accuracy of _CE_PET/CT quantitative values with various contrast enhancements, taking *ex vivo* counting as the gold standard. A large cohort of animals was imaged with various contrast media protocols including intraperitoneal injection of iohexol and intravenous injection of a long-lasting contrast media. Overall, in 43 lesions, a good correlation was found between quantitative values extracted from SA-PET data and *ex vivo* counting, with an *r*^2^ value of 0.87 (*P* < 0.0001). Bland-Altman analysis showed a mean ratio between *ex vivo* counting and SA-PET data of 0.99 (95% confidence interval (CI) 0.69 to 1.31) (Figure 3). Analyzing rat lesions (*n* = 13) and mouse lesions (*n* = 30) separately, Bland-Altman analysis showed that mean ratios between *ex vivo* counting and SA-PET data were 0.98 (95% CI 0.76 to 1.20) and 1.0 (95% CI 0.67 to 1.35), respectively. Analyzing lesions in animals that had received a long-lasting intravascular contrast media injection (Fenestra VC or eXia 160 XL, *n* = 13) and those in animals that had received the liver-selective contrast agent (Fenestra LC, *n* = 30) separately, Bland-Altman analysis showed that mean ratios between *ex vivo* counting and SA-PET data were 1.03 (95% CI 0.96 to 1.11) and 1.02 (95% CI 0.94 to 1.10), respectively.

**Figure 4:**
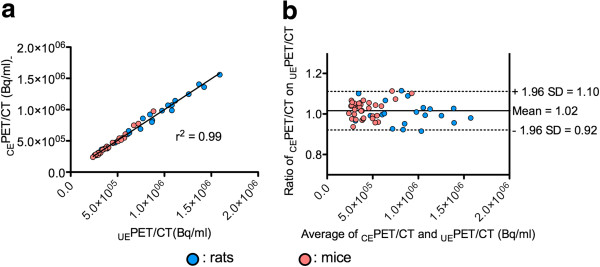
**Impact of high densities induced by iodine-based contrast media on SA-PET/CT quantitative values: animal scans.** Linear regression (**a**) and Bland-Altman analysis (**b**) of SA-PET/CT quantitative values in unenhanced PET/CT (_UE_PET/CT) as compared to contrast-enhanced PET/CT (_CE_PET/CT). Abdominal tumor-bearing nude mice and nude rats were sacrificed after the ^18^F-FDG uptake period, scanned without contrast media, and subsequently scanned after intraperitoneal injection of iohexol (3 mL, 100 mg I/mL).

The second experiment evaluated the impact of intraperitoneal iodinated contrast agent on quantitative values by comparing quantitative values before and after injection of intraperitoneal contrast media. Overall, 55 lesions were analyzed, and an excellent correlation was found between quantitative values extracted from _UE_PET/CT and those extracted from _CE_PET/CT, with an *r*^2^ value greater than 0.9 (*P* < 0.0001). Bland-Altman analysis showed a mean ratio between _CE_PET/CT and _UE_PET/CT quantitative values of 1.02 (95% CI 0.92 to 1.10) (Figure 4). When analyzing rat lesions (*n* = 21) and mouse lesions (*n* = 34) separately, Bland-Altman analysis showed that the mean ratios between _CE_PET/CT and _UE_PET/CT quantitative values were 0.98 (95% CI 0.87 to 1.09) and 1.01 (95% CI 0.92 to 1.10), respectively.

### Impact of contrast media on tumor localization and diagnostic performance

**Figure 5:**
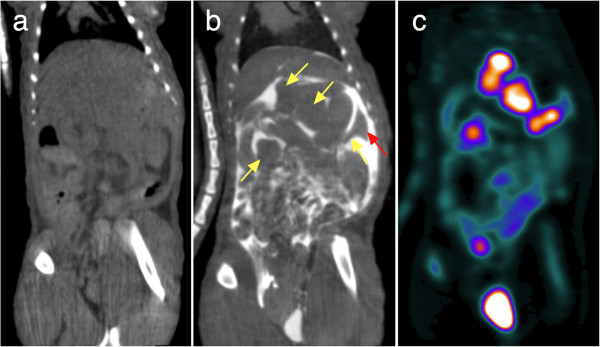
**Impact of intraperitoneal and intravenous contrast media enhancement on tumor localization on SA-CT images: rats.** Representative coronal slices for unenhanced CT (**a**) and contrast-enhanced SA-PET/CT (**b** and **c**) of a rat with multiple abdominal lesions. The animal first underwent SA-CT acquisition, was subsequently injected with a mixture of ^18^F-FDG and Fenestra VC, and received an intraperitoneal injection of iohexol immediately before the SA-PET/CT acquisition began. Tumors are well defined in the contrast-enhanced CT slice (yellow arrows). Also visible are the liver and the spleen (red arrow). Note that due to rapid absorption through the peritoneum, contrast media is excreted in the bladder.

**Figure 6:**
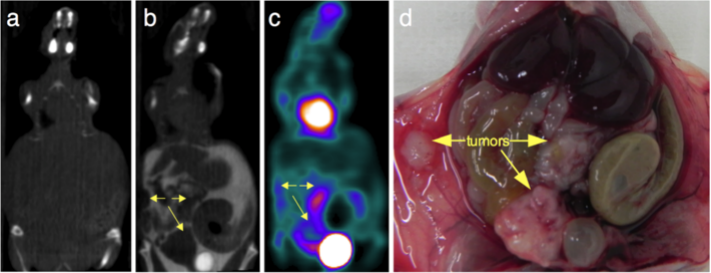
**Impact of intraperitoneal and intravenous contrast media enhancement on tumor localization on SA-CT images: mice.** Representative coronal slices for unenhanced CT (**a**), contrast-enhanced SA-PET/CT (**b** and **c**), and necropsy photograph of a mouse with multiple abdominal lesions and hemorrhagic ascites (**d**). The animal first underwent SA-CT acquisition, was subsequently injected with a mixture of ^18^F-FDG and Fenestra VC, and received an intraperitoneal injection of iohexol immediately before the SA-PET/CT acquisition began. Tumors are well defined on the contrast-enhanced CT slice (yellow arrows), including a necrotic lesion located near the bladder harboring a low ^18^F-FDG uptake and a central photopenic area on an SA-PET slice. Also visible is a tumor at the site of tumor cell injection.

**Figure 7:**
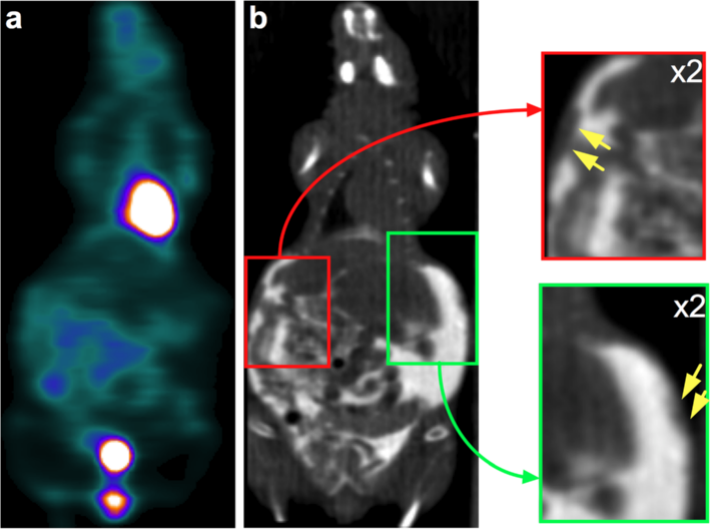
**Example of carcinomatosis lesions depicted by contrast-enhanced CT and overlooked by PET.** Representative coronal slices for ^18^F-FDG SA-PET/CT (**a**) and contrast-enhanced CT (**b**) of a mouse with multiple abdominal lesions. Small carcinomatosis lesions (insets, yellow arrows) involving the abdominal wall are visible on CT but are not ^18^F-FDG avid.

Figures 5 and 6 illustrate how the intravenous injection of a mixture of ^18^F-FDG and iodinated contrast agent combined with intraperitoneal iohexol injection immediately before the start of the SA-PET/CT acquisition improved visual delineation of the intraperitoneal tumor from the surrounding organs in rats and mice. In our experience, this was of particular value in the evaluation of tumors with a heterogeneous tracer uptake that could easily be mistaken for physiological uptake. In addition, this technique facilitates and improves the visual evaluation of the intra-abdominal organs. The kidneys were well visualized because of the urinary elimination of the intravenous iodinated contrast agent and because of an early peritoneal absorption after intraperitoneal injection, leading to the presence of contrast media in the renal collecting systems and bladder. Therefore, the bladder was very well defined both on its inner side by the intravesical accumulation of contrast media eliminated in the urine and on its outer side by the remaining intraperitoneal iodinated agent, allowing for excellent delineation of the bladder wall. The injection of long-lasting contrast agents was also useful in providing additional anatomical landmarks through liver and spleen enhancement. In addition, the contrast-enhanced CT images showed small volume tumoral deposits in one rat and one mouse that were not visualized on the PET images, as shown in Figure 7.

**Figure 8:**
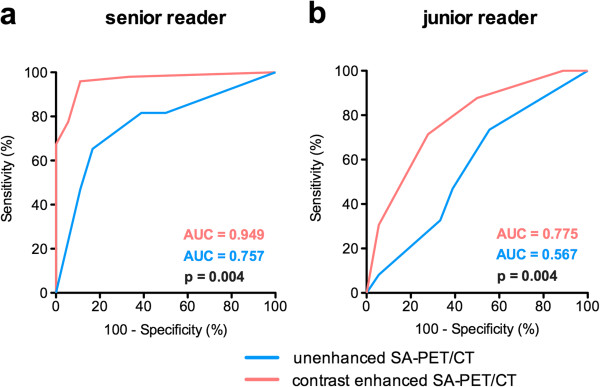
**ROC analysis (a and b) for **_**CE**_**PET/CT and **_**UE**_**PET/CT data.** Each focus was classified according to a 5-point scale (1, definitely benign; 2, probably benign; 3, indeterminate; 4, probably malignant; 5, definitely malignant) for each examination (without and with the use of intraperitoneal contrast media). Final foci status was assessed at necropsy.

Receiver operating characteristic (ROC) analyses were performed to objectively compare the results of _UE_PET/CT and _CE_PET/CT data interpretation according to a 5-point scale (Figure 8). A total of 69 ^18^F-FDG foci were indentified during the review process. Based on the results of the necropsy, 51 abdominal foci corresponded to tumors, while 18 of them were located in animals or regions of the abdomen that were free of disease. For the senior observer, the area under the receiver operating characteristic curve was 0.77 (95% CI 0.65 to 0.86) for _UE_PET/CT and 0.96 (95% CI 0.89 to 0.99) for _CE_PET/CT (*P* = 0.004). For the junior observer, the area under the receiver operating characteristic curve was 0.58 (95% CI 0.45 to 0.70) for _UE_PET/CT and 0.78 (95% CI 0.66 to 0.87) for _CE_PET/CT (*P* = 0.004). When selecting the third level of the scale as the diagnostic threshold, sensitivity and specificity for the senior observer were respectively 65% (95% CI 40 to 78) and 83% (95% CI 59 to 96) for _UE_PET/CT, and 78% (95% CI 63 to 88) and 94% (95% CI 73 to 100) for _CE_PET/CT. Sensitivity and specificity for the junior observer with the same level of scale were respectively 34% (95% CI 21 to 41) and 67% (95% CI 41 to 87) for _UE_PET/CT, and 71% (95% CI 57 to 83) and 72% (95% CI 47 to 90) for _CE_PET/CT.

## Discussion

We performed a comprehensive evaluation of the diagnostic performance and accuracy of SA-PET quantitative values, comparing _CE_PET/CT with _UE_PET/CT in phantom and animal studies including rats and mice with a wide range of abdominal tumors. The use of iodinated contrast media improved tumor delineation on CT images, thus providing better diagnostic accuracy, but it induced a moderate overestimation of SA-PET quantitative data and a slight alteration of NEMA NU 4–2008 image quality parameters.

### Impact of contrast media on NEMA NU 4–2008 image quality parameters and accuracy of quantitative values

Phantom acquisitions involving contrast media were performed at an iodine concentration of 100 mg I/mL, as this concentration results in CT densities much higher than those obtained with long-lasting contrast agents and is thus more likely to alter the attenuation map derived from segmented CT images. NEMA NU 4–2008 image quality phantom data showed that changes in %SD_unif_ and SORs when attenuation or attenuation plus scatter corrections were applied were slightly different when the NEMA NU 4–2008 image quality phantom was filled either with ^18^F-FDG or with ^18^F-FDG and contrast media. This finding, which was confirmed by imaging the NEMA NU 4–2008 image quality phantom twice for each situation, demonstrates that the use of contrast material minimally affected the accuracy of attenuation and scatter corrections. Noticeably, despite slight changes due to the use of iodinated contrast media, observed %SD_unif_ and SORs were in the range of those reported by other labs running the Inveon system [17,24] and can be compared to NEMA NU 4–2008 evaluation of other modern SA-PET systems that have been recently described in details by Goertzen et al. [25]. The homemade phantom acquisitions demonstrated that, on average, a 10.3% overestimation could be expected when imaging a ^18^F-FDG source that mimics a pertinent-sized tumor surrounded by contrast media, whereas measured activity was almost equal to true activity when the source was surrounded by water. This finding was confirmed by animal experiments comparing _CE_PET/CT and _UE_PET/CT quantitative data, for which Bland-Altman analysis showed that the overestimation in a large cohort of 55 abdominal lesions never exceeded 10%. Furthermore, our results are in the range of those observed with clinical PET/CT systems where a 20 ± 1.8% overestimation was reported when filling a NEMA phantom with a contrast concentration usually used in clinical settings (3% solution of Gastrografin™ Bristol-Myers Squibb, Princeton, NJ, USA; 370 mg I/mL) [26]. Regarding clinical studies, oral contrast media have been shown to induce an average error of 4.4 ± 2.8% (max CT value of 520 HU) [27] and stasis of intravenous contrast media in the subclavian vein has been reported to increase maximum standardized uptake value (SUVmax) by 27.1% [14]. Of note are the similar ratios between PET quantitative data and *ex vivo* counting that we observed for the different types of intravenous contrast media. This shows that the overestimation is driven by the intraperitoneal fluid (100 mg I/mL) and that intravenous contrast media can be used without concern on quantitative accuracy.

### Impact of contrast media on diagnostic accuracy

As shown in Figures 5 and 6, intraperitoneal injection of an iodinated contrast agent dramatically improved delineation of an intraperitoneal tumor from the surrounding organs and detection of carcinomatosis involving the abdominal wall or the diaphragm. It is noteworthy that in one mouse and one rat, contrast media also enabled detection of small-volume carcinomatosis on CT that would have been overlooked by PET-only images, as shown in Figure 7. However, this finding was rare and did not discriminate between the presence or absence of carcinomatosis in both animals, as ^18^F-FDG-positive carcinomatosis was also present. The intravenous injection of long-lasting contrast agents was also useful in providing additional anatomical landmarks, thanks to liver and spleen enhancement. This better delineation of tumors was particularly useful in the case of lesions harboring heterogeneous tracer uptake, as shown in Figure 6 in a mouse with a necrotic tumor near the bladder. A new insight from this study is that the fast absorption through the peritoneum leads to the presence of contrast media in the renal collecting systems and in the bladder at higher densities than in the remaining intraperitoneal contrast media, allowing for a clear visualization of the bladder wall. This finding offers new opportunities for imaging bladder cancer models.

An objective assessment of _CE_PET/CT versus _UE_PET/CT images was performed by means of receiver operating characteristic analysis, which showed that _CE_PET/CT performs better than _UE_PET/CT for lesion detection within the abdomen, although in some cases, lesions were correctly identified on _UE_PET/CT images (Figure 8). It is noteworthy that the comparison between _CE_PET/CT and _UE_PET/CT images was made in the group of animals that received only intraperitoneal contrast media; because animals had to be killed after the tracer uptake period to stop the uptake that could have occurred between the two SA-PET examinations, intravenous injection of long-lasting contrast medium was not feasible. Given that liver and spleen enhancement provided by these intravenous contrast media results in useful additional anatomical landmarks, one can assume that the difference in the area under the curve for receiver operating characteristic analysis would have been even more important if both intraperitoneal and intravenous contrast media had been used.

### Should we use only intraperitoneal contrast media or both intraperitoneal and intravenous contrast media?

As shown by receiver operating characteristic analysis, intraperitoneal contrast enhancement with clinical contrast media (iohexol), easily available at low cost, significantly improved diagnostic accuracy and could therefore be used alone. The additional application of long-lasting contrast agents provides further valuable anatomical landmarks but involves a larger volume for intravenous injections and supplemental costs. The choice between intraperitoneal contrast media or a dual-contrast media protocol including intraperitoneal and intravenous contrast agents will be a compromise among cost, potential difficulties for intravenous injection, morbidity, and image quality. Regarding morbidity, to the best of our knowledge, no study has reported side effects due to an intravenous bolus with long-lasting contrast agents in mice or rats.

### Implications when using _CE_PET/CT for therapy-monitoring purposes

SA-PET and SA-PET/CT are being used increasingly in cancer research, with a major interest in the ability to monitor repeatedly and non-invasively the effect of chemotherapy or molecularly targeted therapies. As opposed to clinical PET images that can be interpreted both visually and semi-quantitatively, SA-PET images, when used for therapy monitoring, are interpreted only by means of changes in semi-quantitative values like SUVs or percentage injected dose per gram of tissue (%ID/g). The overestimation of quantitative values due to contrast media, which never exceeded 10% in our study, could be a source of inter-animal variability, previously demonstrated to be 15% on average in mice-bearing tumors imaged twice on the same day, after injection and reinjection of ^18^F-FDG [28], ^18^F-FLT [29], or ^18^F-labeled RGD [30]. Another implication of the use of contrast media is the possible interaction between intraperitoneal contrast media and chemotherapy, molecularly targeted therapies, or vehicle administered intraperitoneally the day of the PET examination. Although the peritoneum is known to allow fast absorption of fluids, further studies are required to investigate the delay required to reach full absorption of intraperitoneal contrast media, as well as potential renal toxicity of repeated intraperitoneal injections, having in mind that some antineoplastic treatments induce kidney damage. The morbidity that could arise from long-lasting agents and that relates to viscosity, volume, and iodine content will also be the topic of a future work.

## Conclusion

This study demonstrates, using a NEMA NU 4–2008 image quality phantom and a homemade phantom, as well as a large cohort of abdominal tumor-bearing mice and rats, that the use of iodinated contrast media for SA-PET imaging significantly improves abdominal tumor delineation and diagnostic performance at the expense of a limited impact on accuracy of quantitative values and image quality parameters.

## Competing interests

The authors declare that they have no competing interests.

## Authors’ contributions

NA conceived the study, participated in its design and coordination and in the measurements, and drafted the manuscript. CL carried out all measurements and data processing and helped draft the manuscript. EQ participated in data analysis and helped draft the manuscript. MB and MHL participated in the study coordination and in the measurements. ZG participated in phantom data analysis and helped draft the manuscript. All authors read and approved the final manuscript.
